# Reinfection of Transplanted Livers in HCV- and HCV/HIV-Infected Patients Is Characterized by a Different MicroRNA Expression Profile

**DOI:** 10.3390/cells11040690

**Published:** 2022-02-16

**Authors:** Emiliano Dalla, Michela Bulfoni, Daniela Cesselli, Riccardo Pravisani, Masaaki Hidaka, Susumu Eguchi, Umberto Baccarani

**Affiliations:** 1 Department of Medicine, University of Udine, 33100 Udine, Italy; emiliano.dalla@uniud.it (E.D.); michela.bulfoni@uniud.it (M.B.); riccardo.pravisani@gmail.com (R.P.); 2 Institute of Pathology, University Hospital of Udine, 33100 Udine, Italy; 3 Liver & Kidney Transplant Unit, University Hospital of Udine, 33100 Udine, Italy; 4 Department of Surgery, Nagasaki University Graduate School of Biomedical Sciences, Nagasaki 852-8523, Japan; mahidaka@dj8-net.ne.jp (M.H.); sueguchi@nagasaki-u.ac.jp (S.E.)

**Keywords:** HCV, HCV/HIV, liver transplantation, miRNA, NGS

## Abstract

Background: After liver transplantation, HCV/HIV co-infected patients present, compared to the HCV mono-infected ones, increased HCV viral load, rapid progression to liver fibrosis and higher mortality. Liver biopsies (LB), obtained routinely 6 months after transplantation, represent a unique model to assess the early events related to graft re-infection. Here, we used miRNA sequencing of LB obtained from both HCV-and HCV/HIV-infected recipients, to identify transcriptional profiles able to explain the more severe outcome of these latter. Methods: miRNAs of 3 healthy livers, 3 HCV-LB and 3 HCV/HIV-LB were sequenced by Illumina HiSeq2500 platform. The DIANA-miRPath v3.0 webserver and DIANA-microT-CDS algorithm (v5.0) were used to characterize the functions of differentially expressed (DE-) miRNAs, querying the KEGG and Gene Ontology-Biological Process databases. Results: LB obtained from infected patients were characterized, with respect to controls, by a miRNA profile related to viral infection, immune system signaling and DNA damage in HCV-induced carcinogenesis. Instead, HCV-LB and HCV/HIV-LB differed in the expression of miRNAs involved in immunological and apoptotic processes and in extracellular matrix remodeling. Conclusions: liver reinfection processes are associated with early miRNA changes. Further studies are necessary to establish their prognostic role and possible actionability.

## 1. Introduction

Since the introduction of highly active antiretroviral therapy (HAART), human immunodeficiency virus type 1 (HIV-1) infection is not, anymore, an exclusion criterion for liver transplantation (LT) in patients with a Hepatitis C virus (HCV)-related end stage disease [1,2,3]. This latter is the consequence of chronic inflammation, immune activation, and immune senescence due to HCV infection [4,5]. However, after transplantation, graft reinfection is a common event and disease progression is even enhanced with respect to non-transplanted patients [5,6]. Despite HAART, in HIV/HCV co-infected patients, the HIV-1 infection has an additional deleterious effect on HCV reactivation in transplanted livers, determining increased viral loads, accelerated progression to liver cirrhosis and higher risk of hepatocellular carcinoma [4]. Some of the pathways that have been proposed to accelerate fibrosis in co-infected patients include direct viral effects, dysregulation of the immune system and other metabolic pathways that lead to liver toxicity and processes such as steatosis and insulin resistance [4].

The introduction of direct-acting antiviral agents (DAAs) against HCV has significantly improved the prognosis of HCV/HIV co-infected patients undergoing liver transplantation for end stage liver disease (ESLD) [7,8,9,10,11]. However, recent studies have suggested that DAA-mediated HCV eradication could represent an impediment to HIV reservoir elimination in coinfected patients [4,12], and that DAA treatment does not completely normalize immune and liver functions as well as the risk of hepatocellular carcinoma development [13,14].

Therefore, it is still fundamental to elucidate the pathogenetic mechanisms underlying the liver damage caused by HCV infection in transplanted livers and the mechanisms by which HIV co-infection can enhance it.

In this regard, microRNAs (miRNAs) are believed to play a major role in the interplay between HIV, HCV and host cells [15,16,17]. MicroRNAs are small, single-stranded, non-coding RNA molecules that act to transcriptionally or post-transcriptionally regulate many different genes, thus, modulating biological processes [18]. During infection, viruses such as HIV can synthesize miRNAs to control their own replication cycle, while host-cell miRNAs can favor or counteract infection by targeting the viral RNA directly or by modulating the expression of cellular genes playing a role in the viral lifecycle [15].

So far, investigations regarding differences in the miRNA profile of HCV- and HCV/HIV co-infected patients were mainly pursued in cirrhotic, explanted livers, and were focused on a priori selected miRNAs [15,16,19]. However, cirrhosis represents the terminal stage of a chronic, irreversible pathologic process with limited possibility of therapeutic interventions. 

Reinfection of human liver grafts represents, instead, a unique and quite unexplored model to study early stages of liver disease and to investigate the possible interaction of HCV and HIV. In this regard, we have recently demonstrated that, as early as 6 months after liver transplantation, grafts of HCV and HCV/HIV recipients differ in the expression of clinically relevant selected miRNAs, suggesting a possible connection between specific miRNA deregulation, viral load and fibrosis [2]. 

The purpose of the present study was to extend the analysis, adopting, for the first time, an untargeted NGS approach. Specifically, we analyzed liver biopsies collected 6 months after liver transplantation from both HCV mono-infected and HCV/HIV co-infected patients, as well as from healthy livers, in order to detect new miRNAs that are either involved in viral infection, inflammation and fibrosis or that are responsible for the more aggressive outcome of liver disease in HCV/HIV coinfected patients.

## 2. Materials and Methods

### 2.1. Sample Collection

NGS analysis was performed in formalin-fixed paraffin-embedded (FFPE) liver biopsies obtained from 3 HCV mono-infected and 3 HCV/HIV co-infected patients. A flow diagram of study sample inclusion and exclusion criteria is depicted in Supplementary Figure S1, while clinicopathological features of selected patients are shown in Supplementary Table S1.

Briefly, LB were routinely collected at 6 months after transplantation as per center-protocol. Specifically, during the 2007–2014 period, 42 HIV/HCV co-infected and 83 HCV mono-infected patients were submitted to liver transplantation (LT) at the Liver Transplant Unit of the University Hospital of Udine. Indications to liver transplantation were identical in HCV mono-infected and HCV/HIV-coinfected patients, as previously reported in [1]. Exclusion criteria included split liver graft, HBV positivity, coexisting autoimmune hepatitis, post-LT surgical or immunologic complications, treatment with DAA-HCV drugs before LT or within 6 months post-LT, unavailability of a liver biopsy obtained at 6 ± 1 months post-LT, a biopsy core containing less than 11 portal spaces, pathologic features of graft rejection or cholangiopathy on liver biopsy [2]. Thus, 19 HCV mono-infected and 20 HCV-HIV coinfected recipients were selected on the basis of the same MELD score, graft steatosis and total ischemia time [10,20]. Recipient age was not considered for the matching because the epidemiology of HCV infection and HCV/HIV co-infection is significantly different in terms of age [2]. Liver biopsies (LB) were formalin-fixed paraffin-embedded (FFPE). LB were re-evaluated by an expert pathologist, according to the Ishak classification, to detect and quantify HCV-related liver fibrosis and inflammation [21]. We selected 6 liver biopsies performed 6 months after liver transplantation of 3 HCV mono-infected and 3 HCV/HIV co-infected recipients characterized by RNA of quantity and quality suitable for RNA sequencing (see below), similar grading and staging Ishak scores. To reduce the preanalytical factors possibly affecting miRNA analysis, all selected biopsies were obtained at the Liver Transplant Center at the University Hospital of Udine, using the same protocol for biopsy sampling, processing and analysis. The average time between sample collection and RNA extraction/library preparation was 121 ± 3 months for HCV LB and 123 ± 5 months for HCV + HIV LB (*p* = 0.5512). As control group, LB from three randomly selected brain deceased donors were used. Inclusion criteria comprised biopsy core containing at least 11 portal spaces, histologically proven normal liver and standard donation with no extended criteria [22]. As mentioned, while LB in the study group (HCV mono-infected and HCV/HIV co-infected liver grafts) were obtained as per-center protocol 6 months after transplantation, LB in the control group (healthy non-infected livers) were obtained from healthy brain deceased liver donors during the warm phase of organ procurement. HCV/HIV negative recipients were not selected as control group because for these patients a systematic post-transplant biopsy was not routinely performed as per protocol. 

Immunosuppression of transplanted patients was centered in all cases on tacrolimus plus steroids and possible mycophenolate mofetil introduction for renal sparing. In HIV recipients, HAART therapy was maintained unmodified after LT. The presence of an active viral replication and the peripheral viral load were tested as per protocol in the preliminary blood tests before the liver biopsy and in all cases the HIV viremia was suppressed and the CD4 lymphocyte count was >200 cells/mmc. Written informed consent was obtained from all participants at the time of LT for the use of FFPE samples for scientific purposes. The present study was approved by the local Institutional Review Board.

### 2.2. RNA Extraction and Library Preparation and Sequencing

Total RNA was extracted from 3 healthy livers, 3 HCV-infected and 3 HCV/HIV co-infected FFPE liver biopsies using the RecoverAll™ Total Nucleic Acid Isolation Kit (Ambion), according to the manufacturer’s instructions. RNA was recovered with 30 μL of nuclease-free water. RNA quantification, quality control, library preparation and sequencing were performed at the IGA Technology Services Srl (https://igatechnology.com). Specifically, the suitability of RNA samples for library preparation was assessed by IGA Technology Services on the basis of RNA quantity, concentration and quality (evaluated as RNA integrity number-RIN-), as measured by Agilent Bioanalyzer 2100. A total amount of 1 μg was analyzed using the “TruSeqSmallRNA Sample Prep kit” (Illumina, San Diego, CA, USA) for library preparation following the manufacturer’s instructions. Both RNA samples and final libraries were quantified by using the Qubit 2.0 Fluorometer (Invitrogen, Carlsbad, CA, USA) and quality tested by the Agilent 2100 Bioanalyzer RNA Nano assay (Agilent technologies, Santa Clara, CA, USA). Libraries were then processed with Illumina cBot for cluster generation on the flowcell, following the manufacturer’s instructions and sequenced on single-end 50 bp mode on HiSeq2500 (Illumina, San Diego, CA, USA). The CASAVA 1.8.2 version of the Illumina pipeline was used to process raw data for both format conversion and de-multiplexing. 

### 2.3. smallRNA Bioinformatics Analysis

Three biological replicates were analyzed for each condition, resulting in nine samples, averaging 12 million sequenced reads per sample. Upon adapter removal with Cutadapt [23] fragments were mapped to the miRbase [24] release 22 database and DE-miRNAs were discovered (abs(logFC) ≥ 1.5) using the DESeq2 R/Bioconductor package [25]. Correction for multiple testing was performed using the Benjamini–Hochberg procedure (padj ≤ 0.05). Limited to the HCV/HIV co-infected versus HCV mono-infected comparison, a *p* < 0.005 data significance threshold was applied to allow the identification of less pronounced differences in miRNA expression.

### 2.4. miRNAs Functional Enrichment Analysis

The DIANA-miRPath v3.0 web-server (Last accessed: November 2018) [26] was used to characterize the functions of differentially expressed human miRNAs based on the predicted miRNA targets provided by the DIANA-microT-CDS algorithm (v5.0). Up- and down-regulated miRNAs were analyzed separately, using the Kyoto Encyclopedia of Genes and Genomes (KEGG) and the Gene-Ontology-Biological Process databases as source of information, applying either a priori (Genes Union) or a posteriori (Pathways/Categories Union) methods. DIANA-miRPath v3.0 extends the Fisher’s exact test, EASE score, and false discovery rate methodologies, with the use of unbiased empirical distributions, to identify the most significantly enriched terms (*p* ≤ 0.05).

## 3. Results

### 3.1. Gene Expression Analyses Highlighted Specific miRNAs Differentially Expressed between Healthy Livers and HCV- as Well as HCV/HIV-Infected Biopsies

Gene expression analysis highlighted 36 miRNAs differentially expressed (DE-miRNAs) between LB obtained from HCV-infected patients (HCV-LB) and from healthy subjects (control-LB) (abs(logFC) ≥ 1.5, padj < 0.05). In particular, with respect to control-LB, HCV-LB were characterized by 21 up-regulated and 15 down-regulated miRNAs (Table 1, left columns and Supplementary Table S2). Eight miRNAs were deregulated in LB from HCV/HIV-infected patients (HCV/HIV-LB) with respect to control-LB (abs(logFC) ≥ 1.5, padj < 0.05): 5 were up-regulated, while 3 were down-regulated (Table 1, central columns and Supplementary Table S2). Finally, comparing miRNAs expression profile of HCV/HIV-LB and HCV-LB, no significant differences were obtained with a padj < 0.05; to overcome this issue, we decided to apply a reduced significant *p*-value (*p* < 0.005) instead of multiple comparison correction. In this way, 15 miRNAs emerged as differentially expressed (abs(logFC) ≥ 1.5, *p* < 0.005): 10 were up-regulated and 5 were down-regulated (Table 1, right columns and Supplementary Table S2).

Interestingly, with the exception of miR-222, miRNAs differentially expressed in HCV/HIV-LB vs. control-LB were similarly regulated (i.e., both up- or down-regulated) in HCV-LB (Table 1, terms shown in italics). Considering, instead, miRNAs up-regulated in HCV/HIV-LB with respect to HCV-LB, three were down-regulated in HCV-LB with respect to controls (Table 1, terms in bold), while the other 7 miRNAs were specific for this comparison.

Altogether, these data confirm the existence of differences in the miRNAs expression profile of LB obtained from HCV- and HCV/HIV-infected patients with respect to control livers, and from HCV/HIV- vs. HCV-infected patients. To understand the meaning of these differences, we performed an in-silico discovery of the gene targets of the DE-miRNAs in order to identify and compare the pathways that are altered during the graft reinfection process in either HCV mono-infected or HCV/HIV co-infected patients.

### 3.2. Comparative Functional Pathway Analyses of the Differentially Expressed miRNAs

Predicted targets of the 36, 8 and 15 DE-miRNAs identified by comparing HCV vs. controls, HCV/HIV vs. controls and HCV/HIV vs. HCV are shown in Supplementary Tables S3–S5. We queried the “Gene Ontology-Biological Process” and the KEGG functional databases to find the significantly enriched functional terms (*p* ≤ 0.05) associated with the predicted target genes.

#### 3.2.1. HCV vs. Controls

**Figure 1:**
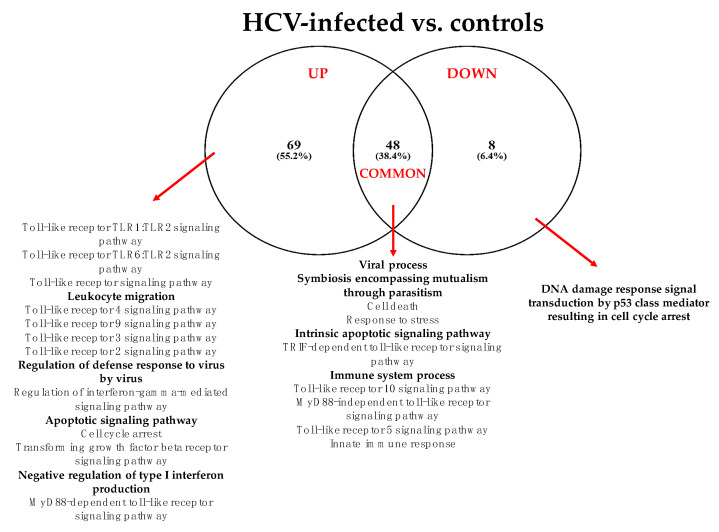
Summary of the “Gene Ontology-Biological Process” enriched functional terms associated with the predicted target genes of miRNAs differentially expressed in LB obtained from HCV-infected patients vs. healthy livers. Some of the most relevant biological processes are shown; the complete lists of enriched terms are available in Supplementary Table S6A.

The summary of the “Gene Ontology-Biological Process” enriched functional terms associated with the 36 DE-miRNAs identified in HCV-LB vs. control-LB is shown in Figure 1 while in Supplementary Tables S6–S8 are reported all the results obtained from both the KEGG and GO analyses.

According to the GO analysis, among the 125 enriched functional terms associated with the predicted target genes of DE-miRNAs, 69 were specific for up-regulated miRNAs, 8 for down-regulated miRNAs and 48 were shared by both miRNA classes. In any case, it was apparent that, 6 months after transplantation, grafts were already characterized by a miRNA profile strictly related to viral infection, immune response, inflammation mediated by chemokine and cytokine signaling, DNA damage and apoptosis. The KEGG analysis additionally indicated the prominent role of DE-miRNAs in cancer-related pathways and prion diseases (Supplementary Tables S6B, S7 and S8). 

**Figure 2:**
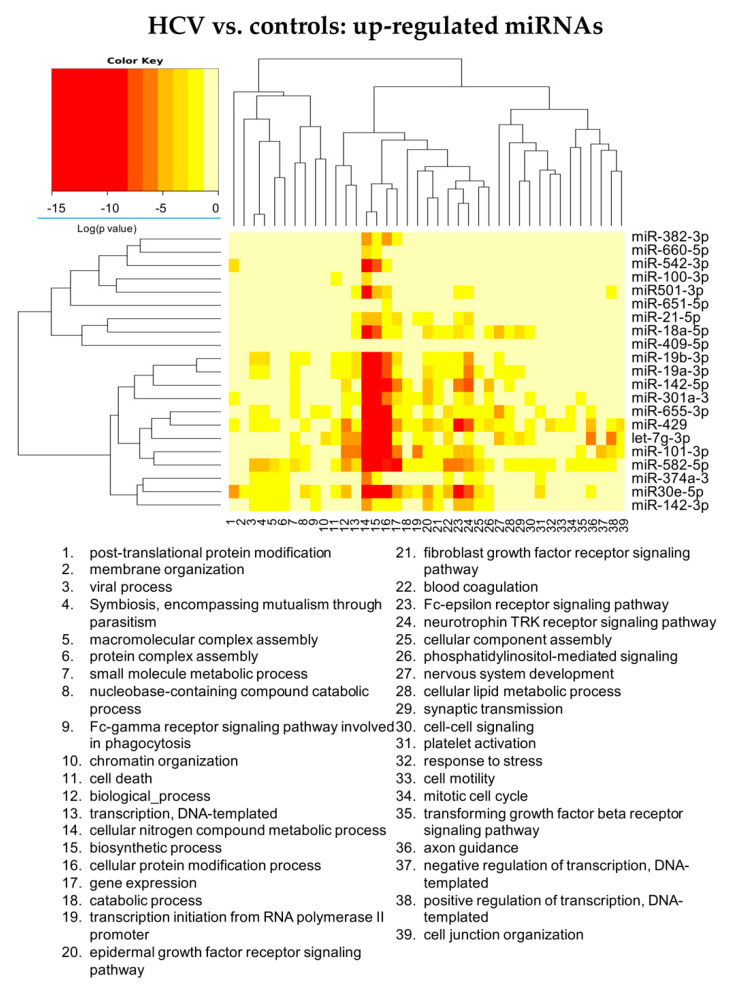
Heatmap showing the significance (see color key) of the association of up-regulated miRNAs with “Gene Ontology-Biological Process” categories, as identified applying the “Categories Union” method.

Analyzing in more details the role of DE-miRNAs in the identified enriched pathways, up-regulated miRNAs can be classified in three groups (Figure 2 and Supplementary Table S7): the first one acts mainly on liver metabolism; the second one involves liver metabolism, cell immunity and cell signaling mediated by tyrosine kinase receptor, including neurothrophin TRK receptor signaling; the third one acts also on viral processes. miRNAs particularly related with cell immunity were miR-30e, miR-429 and miR-142. MiR-19b and miR-582 were associated with pathways related to viral processes. Let-7g was linked with axon guidance. 

**Figure 3:**
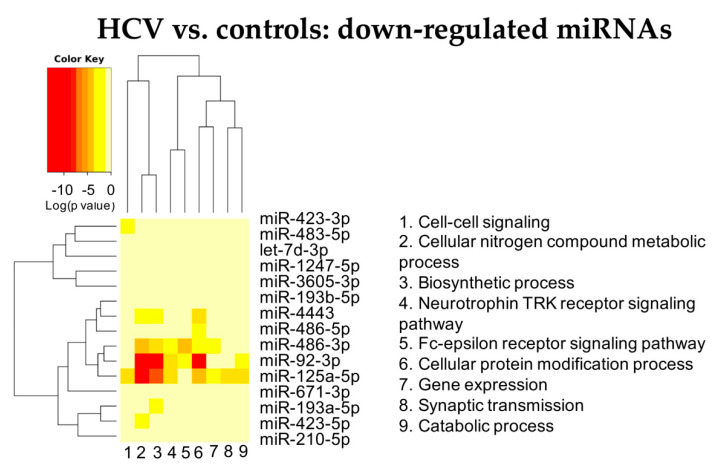
Heatmap showing the significance (see color key) of the association of down-regulated miRNAs with “Gene Ontology-Biological Process” categories, as identified applying the “Categories Union” method.

Among the down-regulated miRNAs, miR-125a, miR-92b and miR486 were also affecting liver metabolism, neurotrophin TRK receptor signaling and cell immunity (Figure 3 and Supplementary Table S8). 

In conclusion, the miRNA profile of grafts, 6 months after transplantation in HCV infected patients, indicates the presence of a profound modification of pathways related to liver metabolism, immunity and activation of tyrosine-kinase-related pathways.

#### 3.2.2. HCV/HIV vs. Controls

**Figure 4:**
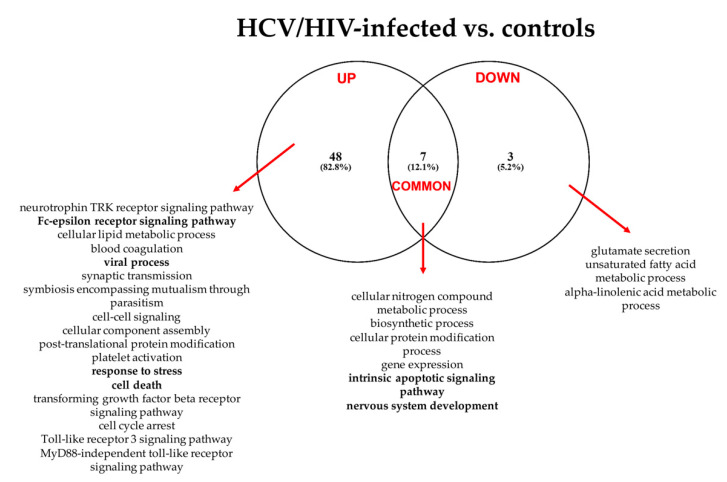
Summary of the “Gene Ontology-Biological Process” enriched functional terms associated with the predicted gene targets of the miRNAs differentially expressed between LB obtained from HCV/HIV co-infected patients vs. healthy livers. Some of the most relevant biological processes are shown; the complete lists of enriched terms are available in Supplementary Table S9A.

Figure 4 summarizes the “Gene Ontology-Biological Process” enriched functional terms associated with the DE-miRNAs identified comparing LB from HCV/HIV co-infected patients with those from healthy livers (see Supplementary Tables S9–S11 for the complete lists of enriched biological processes identified by both the KEGG and GO analyses).

According to the GO analysis, among the 58enriched functional terms associated with the DE-miRNAs, 48 were specific for up-regulated miRNAs, 3 for down-regulated miRNAs and 7 were shared by both miRNA classes.

Similarly to what we observed for the HCV mono-infected patients, in the HCV/HIV co-infected patients, the pathways affected by the DE-miRNAs were linked to viral infection, immune response, inflammation mediated by chemokine and cytokine signaling, DNA damage and apoptosis. In addition, the KEGG analysis indicated the prominent role of DE-miRNAs in cancer-related pathways and prion disease (Supplementary Tables S9B, S10 and S11).

**Figure 5:**
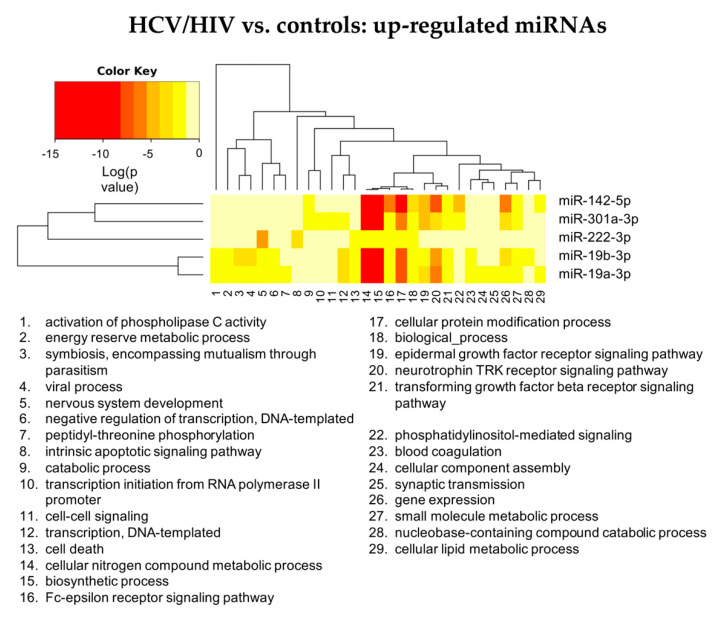
Heatmap showing the significance (see color key) of the association of up-regulated miRNAs with “Gene Ontology-Biological Process” categories, as identified applying the “Categories Union” method.

Analyzing in more details the role of DE-miRNAs in the identified enriched pathways, up-regulated miRNAs can be classified in three groups (Figure 5 and Supplementary Table S10): miR-142 and miR-301a acting mainly on liver metabolism, immunity and cell signaling mediated by tyrosine kinase receptor, including neurothrophin TRK receptor signaling; miR-19a and miR-19b impacting also on viral processes; miR-222 appearing less strongly associated with liver metabolism and immunity but acting on pathways related to intrinsic apoptotic signaling pathways and nervous system development.

**Figure 6:**
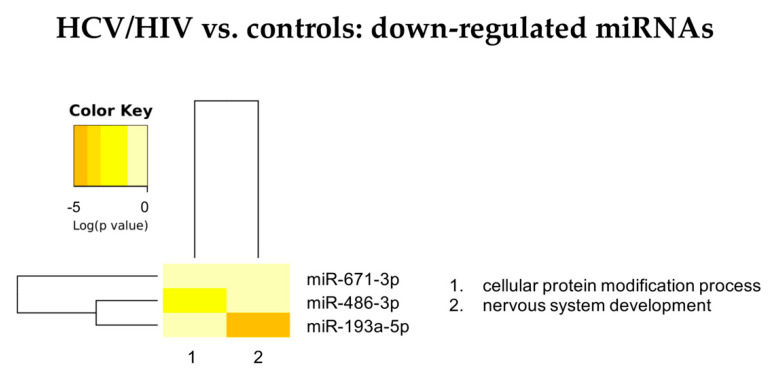
Heatmap showing the significance (see color key) of the association of down-regulated miRNAs with “Gene Ontology-Biological Process” categories, as identified applying the “Categories Union” method.

Among the down-regulated miRNAs (Figure 6 and Supplementary Table S12), miR-193a-5p was affecting nervous system development, too, while miR-486-3p was involved in protein modification processes, similarly to most of the up-regulated miRNAs.

In conclusion, the miRNA profile of grafts, 6 months after transplantation in HCV/HIV co-infected patients, indicates the presence of a profound modification of pathways related to liver metabolism, immunity, viral processes and activation of tyrosin-kinase-related pathways.

#### 3.2.3. HCV/HIV Co-Infected vs. HCV Mono-Infected

**Figure 7:**
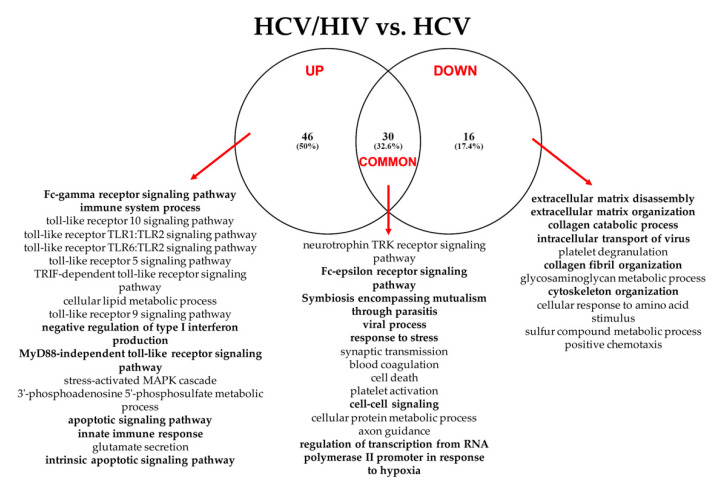
Summary of the “Gene Ontology-Biological Process” enriched functional terms associated with the predicted gene targets of the miRNAs differentially expressed between LB obtained from HCV/HIV co-infected patients vs. HCV mono-infected patients. Some of the most relevant biological processes are shown; the complete lists of enriched terms are available in Supplementary Table S10A.

Figure 7 summarizes the “Gene Ontology-Biological Process” enriched functional terms associated with the DE-miRNAs identified comparing LB from HCV/HIV co-infected patients with those from HCV mono-infected livers (see Supplementary Tables S12–S14 for the complete lists of enriched biological processes identified by both the KEGG and GO analyses).

According to the GO analysis, among the 92 enriched functional terms associated with the DE-miRNAs, 46 were specific for up-regulated miRNAs, 16 for down-regulated miRNAs; 30 were shared by both miRNA classes.

Among these enriched biological processes, we found once again pathways related to cell metabolism, immunity, viral process and blood coagulation, suggesting the existence of different modulatory events occurring in HCV and HCV/HIV patients (Supplementary Tables S13 and S14). More interestingly, miRNAs differentially expressed between mono- and co-infected patients were also regulating genes related to extracellular matrix remodeling and collagen fibril organization. We also observed an increase in terms related to innate immunity, including pathways linked to Fc-gamma receptor signaling, and cell death. The KEGG analysis confirmed the role of DE-miRNAs in extracellular remodeling processes and cancer (Supplementary Tables S12 and S14). 

**Figure 8:**
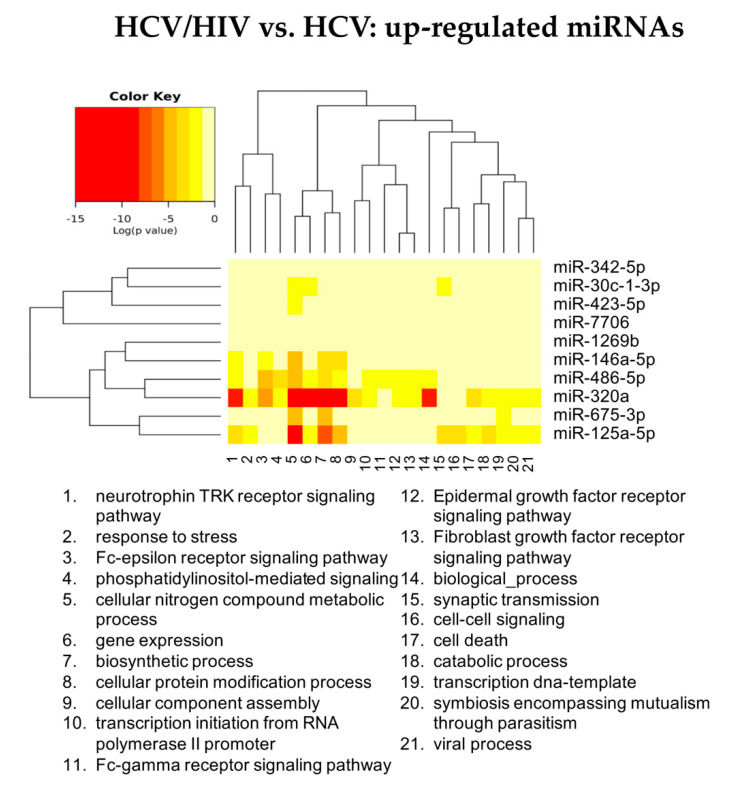
Heatmap showing the significance (see color key) of the association of up-regulated miRNAs with “Gene Ontology-Biological Process” categories, as identified applying the “Categories Union” method.

Analyzing in more details the role of DE-miRNAs in the identified pathways, the up-regulated miR-320a, miR-675and miR-125a possess a pleiotropic activity, since they appeared to regulate the expression of genes linked to all the identified enriched GO categories (Figure 8 and Supplementary Table S13). 

**Figure 9:**
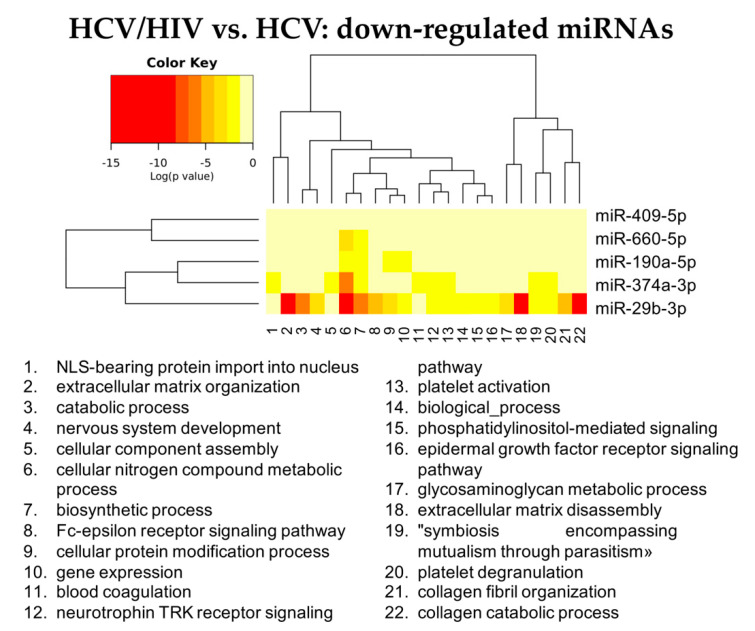
Heatmap showing the significance (see color key) of the association of down-regulated miRNAs with “Gene Ontology-Biological Process” categories, as identified applying the “Categories Union” method.

Conversely, among down-regulated miRNAs, miR-29b was characterized by the widest activity, being strongly associated with the pathways governing extracellular matrix remodeling and collagen catabolism and organization (Figure 9 and Supplementary Table S14). 

Altogether, these data indicate that, after just 6 months, grafts implanted either in HCV or HCV/HIV-infected patients display a miRNA profile associated with the regulation of pathways linked to viral infection, immune response and coagulation. However, in HCV/HIV co-infected patients, it was already possible to identify DE-miRNAs strictly related to extracellular matrix remodeling and immune response to the HIV infection.

## 4. Discussion

The introduction of both HAART for HIV-1 infection and DAAs against HCV has significantly improved the prognosis of HIV/HCV co-infected patients with ESLD [1,2,3,7,8,9,10,11]. However, reinfection of graft is practically inevitable in HCV positive transplanted patients and the presence of co-infection with HIV makes the evolution towards fibrosis more rapid [4]. This effect seems to be mediated by a direct viral effects, dysregulation of the immune system and by metabolic pathways responsible for liver toxicity [4]. 

In humans, the information regarding the synergic effect of HCV and HIV co-infection has been mainly studied in ESLD setting [15,16,19,27]. Here, we have undertaken the issue by introducing two novel elements. First, we studied the reinfection process of liver grafts by taking advantage of 6 months post-transplant protocol LB. Second, we adopted an untargeted NGS approach to define the miRNA profile of liver biopsies. miRNAs are able to regulate many genes simultaneously, making their action on target pathways more incisive [18]. Additionally, they are well-preserved in FFPE tissues, thus, making miRNA expression analysis feasible and reliable [28,29]. 

Comparing healthy livers with LB obtained from transplanted patients, it emerged, that the miRNA profile of liver grafts transplanted in HCV positive patients, either mono-infected or HCV/HIV co-infected, showed an important activation of the innate immunity mechanisms. As a matter of fact, many of the terms emerged from GO and KEGG analyses of predicted target genes were connected to Toll-like receptors related pathways. The TLR family comprises 10 members (TLR1–TLR10) and can recognize both microbe-specific molecular signature (also known as pathogen-associated molecular patterns or PAMPs) and self-derived molecules originated from cell damage (also known as damage-associated molecular patterns or DAMPs). The recognition of PAMPs and DAMPs determines the recruitment of TIR domain-containing adaptor proteins (e.g., MyD88 and TRIF) with the subsequent activation of NF-κB, IRFs, or MAP kinases, thus, modulating the expression of cytokines, chemokines, and type I IFNs to counteract infection [30]. All these pathways were targeted by DE-miRNAs; the role of TLR in liver disease has been already explored, both in HCV mono-infected and HCV/HIV co-infected patients [31,32,33]. DE-miRNAs are additionally targeting both Fc-gamma receptor signaling pathways involved in phagocytosis and Fc-epsilon receptor signaling pathway, thus, further connecting the adaptive immune response to the innate one. 

It is interesting to note that the number of DE-miRNAs impinging on immunity was lower in HCV/HIV co-infected patients with respect to the mono-infected ones. miRNAs known to counteract viral infection and cancer development, such as miR30e [34,35], let-7g [36], miR-429 [37,38] and miR-582 [38], were indeed upregulated in HCV-LB but not in HCV/HIV-LB. Whether this can indicate a blunted antiviral response in HCV/HIV co-infected grafts, responsible for the increased HCV replication present in co-infected patients [39], remains to be demonstrated. Nonetheless, only HCV/HIV co-infected grafts showed an up-regulation of miR-222, known to play a role in the inhibition of HIV entry in macrophages by targeting CD4 [40]. miR-222 is also a well-recognized oncogenic miRNA [41], known to play a role in hepatocellular carcinoma (HCC), by acting on AKT signaling [42,43]. Additionally, it contributes to liver fibrosis by activating stellate cells [44,45]. Interestingly, it has been displayed that circulating levels of miR-222 correlate with HCC stage and prognosis [41,42,43], suggesting that, also in transplanted patients, miR-222 plasma levels could play a role as biomarkers.

Three unexpected pathways resulted to be influenced by DE-miRNAs: coagulation/hemostasis, central nervous system (CNS) disorders and prionic disease. A potential pathophysiological explanation may be found in the exosome mechanism of action. Exosomes are extracellular vesicles of endocytic origin that are used by remote cells as carriers of signaling molecules in an intercellular communication system. The mediators transported by exosomes comprise not only proteins and lipids but also mRNA and miRNA [46,47]. It has been demonstrated that several pathological conditions can modify either the number or the biological content of exosomes, thus, contributing to local and distant manifestations of the disease [46,47].

It is known that HCV infection is associated with an increased thrombotic risk, especially in portal vein system [48], while HIV infection with a recurrent venous thromboembolism [49]. HCV virus can contribute to thrombosis by endothelial damage and tissue factor activation, increased risk of thrombin formation and hypercoagulability. However, more recently, it has been demonstrated that in HCV infection, there is an increased release of exosomes whose miRNA profile is associated with the coagulation cascade and platelet activation [50,51,52]. Similarly, plasma exosomes of HIV patients are characterized by molecular content able to modulate immune activation and inflammation [53,54]. The present data suggest the intriguingly hypothesis that infected liver cells can release into the bloodstream exosomes characterized by a miRNA content able to contribute to the increased thrombotic risk characterizing HCV and HIV infection. 

Concerning the association of HCV and HIV with neurological disorders, it is accepted that the HCV viral genome could prompt demyelination via an immuno-mediate response and that endothelial cells could play a critical role in the transit of HVC into the CNS and promote HCV infection of microglia and astrocytes [55]. Infected macrophages and microglia cells could release pro-inflammatory cytokines, such as TNF-α, IL-1, and IL-6, neurotoxins such as nitric oxide, and viral proteins, which could induce an alteration in brain function [56]. Clinically, HCV patients may most frequently present with symmetrical axonal sensory or sensory-motor polyneuropathy, as well as neurocognitive disorders, which are pathophysiologically independent from liver cirrhosis and tend not to improve after DAA treatment [55]. HIV infection is also associated with neurological disorders and, in case of co-infection, can facilitate HCV replication not only in the liver but also in extrahepatic sites, probably for the general immunosuppression [57]. In fact, co-infected patients showed higher cognitive impairment than patients with exclusive HIV infection [58]. In this regard, it has been demonstrated that the miRNA content of circulating exosomes can play a role in neurological disorders in both HIV [59] and HCV patients [60]. 

Finally, considering the relationship between miRNA profile and prionic diseases, it has been shown that, in hepatocytes infected by HCV, there is an upregulation of host prion protein (PrP) able to facilitate HCV replication [61]. PrP up-regulation was assessed also in astrocytes and neurons of HIV-infected patients suffering from neurological disorders [62].

After establishing the role of DE-miRNAs between HCV- and HCV/HIV-LB with respect to healthy livers, we compared the miRNA profile of HCV-LB with HCV/HIV-LB, applying a reduced significant *p*-value (*p* < 0.005) instead of multiple comparison correction, in order to identify less pronounced differences in miRNA expression. HCV/HIV-LB patients were characterized by the upregulation of miRNAs possibly interfering with the immune response to HCV. In fact, miR-125a targets mitochondrial antiviral signaling and TNF receptor associated factor 6, thus, compromising type I IFN response to HCV and facilitating its replication [63]. Less clear is the role of the up-regulation of miR-486 and miR-320. These miRNAs play an oncosuppressive role and are usually down regulated in HCV infected patients [64,65]. 

More interestingly, the down regulation, in HCV/HIV-LB, of miRNAs implicated in extra cellular matrix remodeling, such as miR29-3p, miR374a-3p, miR660, miR190a and miR409, could be responsible for the early onset of fibrosis in double-infected transplanted livers. In particular, the downregulation of miR29b has been associated to liver fibrosis in several human and murine models [66]. The miR-29 family modulate several signaling pathways linked to progression of hepatic fibrosis, such as NF-κB signaling pathway, Hh signaling pathway, TGF-β signaling pathway and PI3K/AKT signaling pathway [67]. Furthermore, downregulation of miR-29b is associated with cancer development and progression in numerous tumors, including HCC [68]. Since its crucial role, miR-29b is considered a promising biomarker for liver disease and HCC [69]. Moreover, since miR-29b is involved in many extrahepatic pathological conditions, therapies aimed at reestablishing miR-29b expression are currently under evaluation [70,71,72,73]. It is worth mentioning a recent paper in which authors showed, by sequencing of circulating miRNAs, the possibility to early predict liver fibrosis in HCV/HIV [74]. 

This paper presents several limitations. The retrospective modality of data analysis did not allow to undertake either a specific clinical assessment of patients’ neurological and neurocognitive function, nor any analysis on blood levels and content of exosomes. The number of samples analyzed is limited. This is the result of an attempt to reduce as much as possible preanalytical and clinicopathological factors possibly affecting miRNA analysis, as depicted in Supplementary Figure S1. Our objective was to obtain homogeneous groups to test, with a deep-sequencing approach, differentially expressed miRNAs. Unfortunately, the need to have RNA of quantity and quality appropriate for RNA sequencing further reduced the number of suitable samples. However, we compared biological triplicates of the examined conditions, applying, as much as possible, the most stringent statistical thresholds to guarantee a high statistical power. Moreover, deceased donors, as control group, may not be appropriately comparable with the study groups due to the brain death status and no active immunosuppression therapy. Further studies using a larger sample size and including HCV/HIV negative LT recipients as control group will probably be warranted. 

In conclusion, 6 months after transplantation in HCV mono-infected or HCV/HIV co-infected patients, liver grafts display a miRNA profile indicating the activation of functional networks associated with the processes of viral reinfection and, unexpectedly, of neurologic impairment and coagulation disorders. Additionally, in HCV/HIV coinfected patients, an early deregulation of miR-222 and miR29b could play a crucial role in impairing the immune response and in favoring fibrosis. The possibility to modulate the miRNA profile could represent, in the future, a novel therapeutic strategy.

## Figures and Tables

**Table 1:** List of miRNAs differentially expressed between liver biopsies obtained from: HCV mono-infected patients vs. controls (left columns), HCV/HIVco-infected patients vs. controls (central columns) and HCV/HIV co-infected patients vs. HCV mono-infected patients (right columns). In italics we show miRNAs similarly regulated in HCV- and HCV/HIV-infected patients with respect to controls.

HCV Mono-Infected vs. Controls		HCV/HIV Co-Infected vs. Controls		HCV/HIV Co-Infected vs. HCV Mono-Infected	
Up Regulated	Down Regulated	Up Regulated	Down Regulated	Up Regulated	Down Regulated
hsa-miR-18a-5p	hsa-let-7d-3p	*hsa-miR-301a-3p*	*hsa-miR-193b-5p*	hsa-miR-675-3p	hsa-miR-409-5p
hsa-miR-382-3p	hsa-miR-423-3p	*hsa-miR-142-5p*	*hsa-miR-671-3p*	hsa-miR-1269b	hsa-miR-660-5p
hsa-miR-651 5p	*hsa-miR-193a-5p*	*hsa-miR-19a-3p*	*hsa-miR-486-3p*	hsa-miR-342-5p	hsa-miR-190a-5p
hsa-miR-655-3p	hsa-miR-483-5p	hsa-miR-222-3p		hsa-miR-7706	hsa-miR-29b-3p
*hsa-miR-301a-3p*	**hsa-miR-423-5p**	*hsa-miR-19b-3p*		hsa-miR-30c-1-3p	hsa-miR-374a-3p
hsa-let-7g-3p	hsa-miR-92b-3p			**hsa-miR-423-5p**	
hsa-miR-100-3p	*hsa-miR-193b-5p*			**hsa-miR-125a-5p**	
hsa-miR-409-5p	*hsa-miR-671-3p*			hsa-miR-320a-3p	
hsa-miR-374a-3p	**hsa-miR-486-5p**			**hsa-miR-486-5p**	
*hsa-miR-19a-3p*	**hsa-miR-125a-5p**			hsa-miR-146a-5p	
*hsa-miR-19b-3p*	hsa-miR-210-5p				
*hsa-miR-142-5p*	*hsa-miR-486-3p*				
hsa-miR-582-5p	hsa-miR-1247-5p				
hsa-miR-429	hsa-miR-3605-3p				
hsa-miR-660-5p	hsa-miR-4443				
hsa-miR-21-5p					
hsa-miR-542-3p					
hsa-miR-142-3p					
hsa-miR-101-3p					
hsa-miR-501-3p					
hsa-miR-30e-5p					

## Data Availability

Data is contained within the article or supplementary material. Any additional information is available on reasonable request from the corresponding author.

## Supplementary Materials

The following are available online at https://www.mdpi.com/article/10.3390/cells11040690/s1: Supplementary Figure S1: Flow diagram of study sample inclusion and exclusion criteria. Supplementary Table S1: Clinicopathological features of the 6 cases included in the study. LB: liver biopsy. Supplementary Table S2: A. List of differentially expressed miRNAs profiled in HCV mono-infected versus healthy subjects. Only transcripts significantly up-regulated (*n* = 21) or down-regulated (*n* = 15) are shown (abs(log2FC) ≥ 1.5, padj < 0.05); B. List of differentially expressed miRNAs profiled in HCV/HIV co-infected versus healthy subjects. Only transcripts significantly up-regulated (*n* = 5) or down-regulated (*n* = 3) are shown (abs(log2FC) ≥ 1.5, padj < 0.05); C. List of differentially expressed miRNAs profiled in HCV/HIV co-infected versus HCV mono-infected subjects. Only transcripts significantly up-regulated (*n* = 10) or down-regulated (*n* = 5) are shown (abs(log2FC) ≥ 1.5, *p* < 0.005). Values are ordered by decreasing log2FC. Supplementary Table S3: A. Target genes of the 21 miRNAs upregulated in liver biopsies obtained from HCV mono-infected patients with respect to controls, as provided by the DIANA-microT-CDS algorithm; B. Target genes of the 15 miRNAs downregulated in liver biopsies obtained from HCV mono-infected patients with respect to controls, as provided by the DIANA-microT-CDS algorithm. Supplementary Table S4: A. Target genes of the 5 miRNAs upregulated in liver biopsies obtained from HCV/HIV co-infected patients with respect to controls, as provided by the DIANA-microT-CDS algorithm; B. Target genes of the 3 miRNAs downregulated in liver biopsies obtained from HCV/HIV co-infected patients with respect to controls, as provided by the DIANA-microT-CDS algorithm. Supplementary Table S5: A. Target genes of the 10 miRNAs upregulated in liver biopsies obtained from HCV/HIV co-infected patients with respect to those obtained from HCV mono-infected ones, as provided by the DIANA-microT-CDS algorithm; B. Target genes of the 5 miRNAs downregulated in liver biopsies obtained from HCV/HIV co-infected patients with respect to those obtained from HCV mono-infected ones, as provided by the DIANA-microT-CDS algorithm. Supplementary Table S6: A. Venn diagram of the “Gene Ontology-Biological Process” (Genes Union) enriched functional terms associated with the predicted target genes of miRNAs up-regulated (GO_UP) or down-regulated (GO_DOWN) in LB obtained from HCV-infected patients vs. healthy livers. Below, the table indicates the list of the “Gene Ontology—Biological Process” enriched functional terms associated with the genes linked only to the miRNAs up-regulated (Only_UP), only to the miRNA downregulated (Only_DOWN) and to both down-regulated and up-regulated miRNAs (Common) in HCV-infected patients vs. healthy livers applying a priori method (Genes Union). B. Venn diagram of the “Kyoto Encyclopedia of Genes and Genomes” (KEGG, Genes Union) enriched functional terms associated with the predicted target genes of miRNAs up-regulated (KEGG_UP) or down-regulated (KEGG_DOWN) in LB obtained from HCV-infected patients vs. healthy livers. Below, the table indicates the list of the “Kyoto Encyclopedia of Genes and Genomes” enriched functional terms associated with the genes linked only to the miRNAs up-regulated (Only_UP), only to the miRNA downregulated (Only_DOWN) and to both down-regulated and up-regulated miRNAs (Common) in HCV-infected patients vs. healthy livers applying a priori method (Genes Union). Supplementary Table S7: A. Summary of the “Gene Ontology-Biological Process” (Genes Union) enriched functional terms associated with the predicted target genes of miRNAs up-regulated in HCV-infected patients vs. healthy livers; B. Summary of the “Gene Ontology-Biological Process” (Categories Union) enriched functional terms associated with the predicted target genes of miRNAs up-regulated in HCV-infected patients vs. healthy livers; C. Summary of the “Kyoto Encyclopedia of Genes and Genomes” (KEGG, Genes Union) enriched functional terms associated with the predicted target genes of miRNAs up-regulated in HCV-infected patients vs. healthy livers; D. Summary of the “Kyoto Encyclopedia of Genes and Genomes” (KEGG, Pathways Union) enriched functional terms associated with the predicted target genes of miRNAs up-regulated in HCV-infected patients vs. healthy livers. Supplementary Table S8: A. Summary of the “Gene Ontology-Biological Process” (Genes Union) enriched functional terms associated with the predicted target genes of miRNAs down-regulated in HCV-infected patients vs. healthy livers; B. Summary of the “Gene Ontology-Biological Process” (Categories Union) enriched functional terms associated with the predicted target genes of miRNAs down-regulated in HCV-infected patients vs. healthy livers; C. Summary of the “Kyoto Encyclopedia of Genes and Genomes” (KEGG, Genes Union) enriched functional terms associated with the predicted target genes of miRNAs down-regulated in HCV-infected patients vs. healthy livers; D. Summary of the “Kyoto Encyclopedia of Genes and Genomes” (KEGG, Pathways Union) enriched functional terms associated with the predicted target genes of miRNAs down-regulated in HCV-infected patients vs. healthy livers. Supplementary Table S9: A. Venn diagram of the “Gene Ontology-Biological Process” (Genes Union) enriched functional terms associated with the predicted target genes of miRNAs up-regulated (GO_UP) or down-regulated (GO_DOWN) in LB obtained from HCV/HIV co-infected patients vs. healthy livers. Below, the table indicates the list of the “Gene Ontology—Biological Process” enriched functional terms associated with the genes linked only to the miRNAs up-regulated (Only_UP), only to the miRNA downregulated (Only_DOWN) and to both down-regulated and up-regulated miRNAs (Common) in HCV/HIV co-infected patients vs. healthy livers applying a priori method (Genes Union). B. Venn diagram of the “Kyoto Encyclopedia of Genes and Genomes” (KEGG, Genes Union) enriched functional terms associated with the predicted target genes of miRNAs up-regulated (KEGG_UP) or down-regulated (KEGG_DOWN) in LB obtained from HCV/HIV co-infected patients vs. healthy livers. Below, the table indicates the list of the “Kyoto Encyclopedia of Genes and Genomes” enriched functional terms associated with the genes linked only to the miRNAs up-regulated (Only_UP), only to the miRNA downregulated (Only_DOWN) and to both down-regulated and up-regulated miRNAs (Common) in HCV/HIV co-infected patients vs. healthy livers applying a priori method (Genes Union). Supplementary Table S10: A. Summary of the “Gene Ontology-Biological Process” (Genes Union) enriched functional terms associated with the predicted target genes of miRNAs up-regulated in HCV/HIV co-infected patients vs. healthy livers; B. Summary of the “Gene Ontology-Biological Process” (Categories Union) enriched functional terms associated with the predicted target genes of miRNAs up-regulated in HCV/HIV co-infected patients vs. healthy livers; C. Summary of the “Kyoto Encyclopedia of Genes and Genomes” (KEGG, Genes Union) enriched functional terms associated with the predicted target genes of miRNAs up-regulated in HCV/HIV co-infected patients vs. healthy livers; D. Summary of the “Kyoto Encyclopedia of Genes and Genomes” (KEGG, Pathways Union) enriched functional terms associated with the predicted target genes of miRNAs up-regulated in HCV/HIV co-infected patients vs. healthy livers. Supplementary Table S11: A. Summary of the “Gene Ontology-Biological Process” (Genes Union) enriched functional terms associated with the predicted target genes of miRNAs down-regulated in HCV/HIV co-infected patients vs. healthy livers; B. Summary of the “Gene Ontology-Biological Process” (Categories Union) enriched functional terms associated with the predicted target genes of miRNAs down-regulated in HCV/HIV co-infected patients vs. healthy livers; C. Summary of the “Kyoto Encyclopedia of Genes and Genomes” (KEGG, Genes Union) enriched functional terms associated with the predicted target genes of miRNAs down-regulated in HCV/HIV co-infected patients vs. healthy livers; D. Summary of the “Kyoto Encyclopedia of Genes and Genomes” (KEGG, Pathways Union) enriched functional terms associated with the predicted target genes of miRNAs down-regulated in HCV/HIV co-infected patients vs. healthy livers. Supplementary Table S12: A. Venn diagram of the “Gene Ontology-Biological Process” (Genes Union) enriched functional terms associated with the predicted target genes of miRNAs up-regulated (GO_UP) or down-regulated (GO_DOWN) in LB obtained from HCV/HIV co-infected patients vs.HCV mono-infected livers. Below, the table indicates the list of the “Gene Ontology—Biological Process” enriched functional terms associated with the genes linked only to the miRNAs up-regulated (Only_UP), only to the miRNA downregulated (Only_DOWN) and to both down-regulated and up-regulated miRNAs (Common) in HCV/HIV co-infected patients vs. HCV mono-infected patients applying a priori method (Genes Union). B. Venn diagram of the “Kyoto Encyclopedia of Genes and Genomes” (KEGG, Genes Union) enriched functional terms associated with the predicted target genes of miRNAs up-regulated (KEGG_UP) or down-regulated (KEGG_DOWN) in LB obtained from HCV/HIV co-infected patients vs. HCV mono-infected livers. Below, the table indicates the list of the “Kyoto Encyclopedia of Genes and Genomes” enriched functional terms associated with the genes linked only to the miRNAs up-regulated (Only_UP), only to the miRNA downregulated (Only_DOWN) and to both down-regulated and up-regulated miRNAs (Common) in HCV/HIV co-infected patients vs. HCV mono-infected patients applying a priori method (Genes Union). Supplementary Table S13: A. Summary of the “Gene Ontology-Biological Process” (Genes Union) enriched functional terms associated with the predicted target genes of miRNAs up-regulated in HCV/HIV co-infected patients vs. HCV mono-infected livers; B. Summary of the “Gene Ontology-Biological Process” (Categories Union) enriched functional terms associated with the predicted target genes of miRNAs up-regulated in HCV/HIV co-infected patients vs. HCV mono-infected livers; C. Summary of the “Gene Ontology-Biological Process” (Categories Union) enriched functional terms associated with the predicted target genes of miRNAs up-regulated in HCV/HIV co-infected patients vs. HCV mono-infected livers; D. Summary of the “Kyoto Encyclopedia of Genes and Genomes” (KEGG, Pathways Union) enriched functional terms associated with the predicted target genes of miRNAs up-regulated in HCV/HIV co-infected patients vs. HCV mono-infected livers. Supplementary Table S14: A. Summary of the “Gene Ontology-Biological Process” (Genes Union) enriched functional terms associated with the predicted target genes of miRNAs down-regulated in HCV/HIV co-infected patients vs. HCV mono-infected livers; B. Summary of the “Gene Ontology-Biological Process” (Categories Union) enriched functional terms associated with the predicted target genes of miRNAs down-regulated in HCV/HIV co-infected patients vs. HCV mono-infected livers; C. Summary of the “Gene Ontology-Biological Process” (Categories Union) enriched functional terms associated with the predicted target genes of miRNAs down-regulated in HCV/HIV co-infected patients vs. HCV mono-infected livers; D. Summary of the “Kyoto Encyclopedia of Genes and Genomes” (KEGG, Pathways Union) enriched functional terms associated with the predicted target genes of miRNAs down-regulated in HCV/HIV co-infected patients vs. HCV mono-infected livers.
