# Supplementary material for: Reinfection of Transplanted Livers in HCV- and HCV/HIV-Infected Patients Is Characterized by a Different MicroRNA Expression Profile
Source: Cells. 2022 Feb 16;11(4):690. doi: 10.3390/cells11040690 (PMC8869900; doi:10.3390/cells11040690)
Supplement: Supplementary file 1 [file cells-11-00690-s001.zip › cells-1549615-supplementary/Supplementary Figure S1.pptx]

## Slide 1
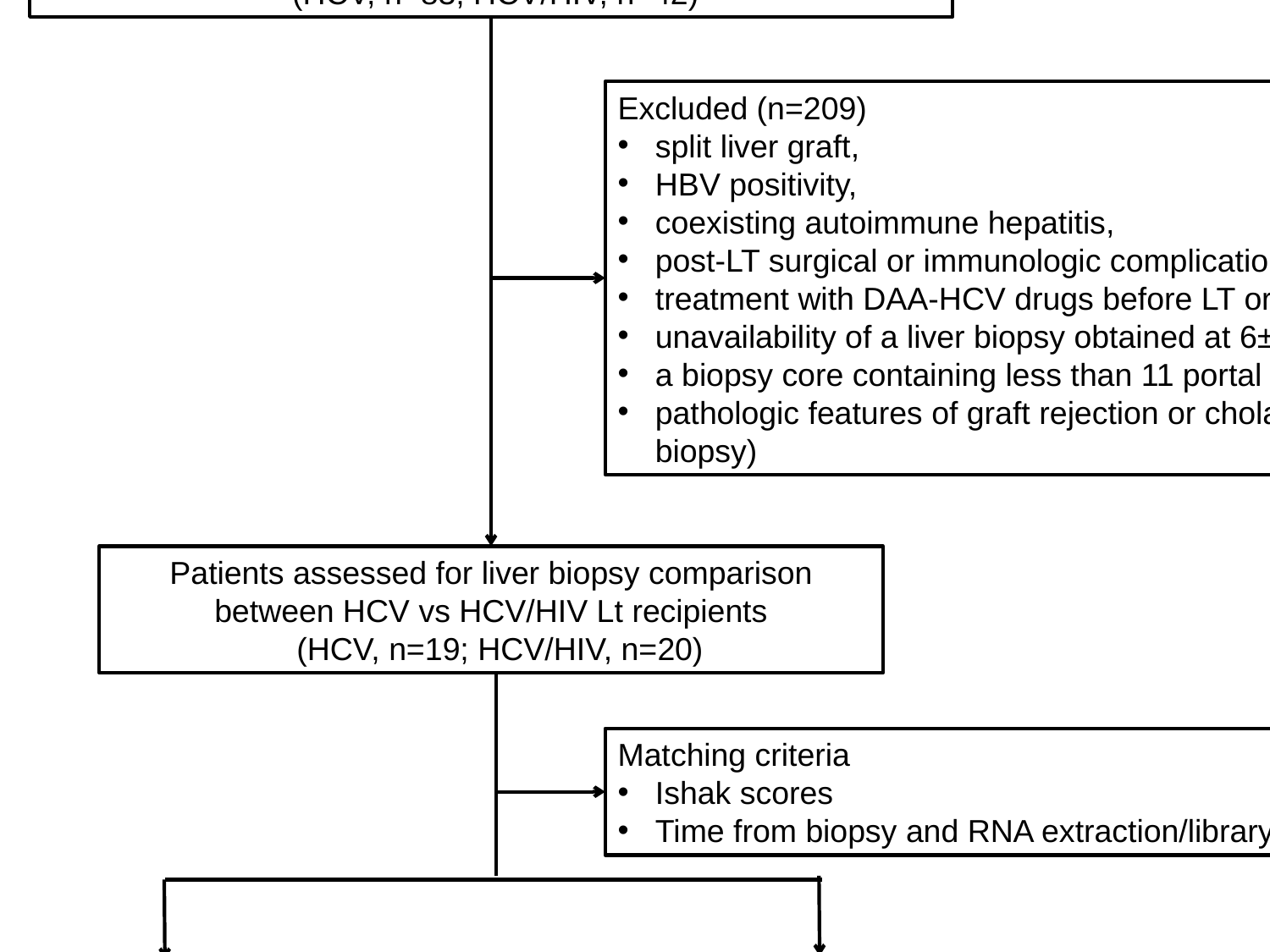

Deceased-donor LT adult recipients
with HCV or HCV/HIV infection
between January 2007 and December 2014
 (HCV, n=83; HCV/HIV, n=42)
Excluded (n=209)
split liver graft,
HBV positivity,
coexisting autoimmune hepatitis,
post-LT surgical or immunologic complications,
treatment with DAA-HCV drugs before LT or within 6 months post-LT,
unavailability of a liver biopsy obtained at 6±1 months post-LT,
a biopsy core containing less than 11 portal spaces,
pathologic features of graft rejection or cholangiopathy on liver biopsy)
Patients assessed for liver biopsy comparison between HCV vs HCV/HIV Lt recipients
 (HCV, n=19; HCV/HIV, n=20)
Matching criteria
Ishak scores
Time from biopsy and RNA extraction/library preparation
HCV/HIV group
 (n=5)
HCV group
 (n=6)
Exclusion criteria
RNA quantity/quality unsuitable for RNA sequencing
HCV/HIV group
 (n=3)
HCV/HIV group
 (n=3)
# Supplementary Figure S1: Flow diagram of study sample inclusion and exclusion criteria
