# Supplementary material for: Reinfection of Transplanted Livers in HCV- and HCV/HIV-Infected Patients Is Characterized by a Different MicroRNA Expression Profile
Source: Cells. 2022 Feb 16;11(4):690. doi: 10.3390/cells11040690 (PMC8869900; doi:10.3390/cells11040690)
Supplement: Supplementary file 1 [file cells-11-00690-s001.zip › cells-1549615-supplementary/Supplementary Table S1.pdf]

Supplementary Table S1. Clinicopathological features of the 6 cases included in the study. LB: liver biopsy.

	HCV			HCV/HIV		
	Patient 1	Patient 2	Patient 3	Patient 1	Patient 2	Patient 3
Age at transplant (years)	69	50	58	44	51	50
Sex	male	male	male	male	male	male
Blood type	A+	AB+	A+	0-	0+	0+
HCV genotype	1B	1A	1A	1A	1B	1A
Donor age (years)	43	76	74	58	46	53
Donor sex	female	male	male	female	female	male
ABO histocompatibility	isocompatible	isocompatible	isocompatible	isocompatible	isocompatible	isocompatible
HCV peripheral viral load, at LB (UI/mL)	1062 $x10^3$	7393 $x10^3$	3917 $x10^3$	6421 $x10^3$	18880 $x10^3$	10460 $x10^3$
HIV peripheral viral load, at LB (UI/mL)	//	//	//	undetectable	undetectable	undetectable
CD4+ count, at LB (cells/uL)	//	//	//	209	621	201
Grading, according Ishak classificantion	4	3	4	4	4	3
Staging, according Ishak classificantion	0	1	1	1	0	1
Time from LB and miRNA analysis (months)	125	121	118	125	118	128
